# Altered Gut Microbiota in Myasthenia Gravis

**DOI:** 10.3389/fmicb.2018.02627

**Published:** 2018-11-09

**Authors:** Dongxu Qiu, Zhiwei Xia, Xiao Jiao, Jun Deng, Lei Zhang, Jing Li

**Affiliations:** ^1^Department of Neurology, Xiangya Hospital, Central South University, Changsha, China; ^2^Key Laboratory of Neurology of Hunan Province, Changsha, China

**Keywords:** myasthenia gravis, 16S rRNA, gut microbiota, *Clostridium*, short-chain fatty acids

## Abstract

Myasthenia gravis (MG) is an autoimmune-mediated disorder, the etiology of which involves both environmental factors and genetics. While the exact factors responsible for predisposition to MG remain elusive, it is hypothesized that gut microbiota play a critical role in the pathogenesis of MG. This study investigated whether gut microbiota are altered in MG patients by comparing the fecal microbiota profiles of MG patients to those of age- and sex-matched healthy controls. Phylotype profiles of gut microbial populations were generated using hypervariable tag sequencing of the V4 region of the 16S ribosomal RNA gene. Fecal short-chain fatty acids (SCFAs) were assessed by gas chromatographic analyses. The results demonstrated that, compared to the healthy cohort, the gut microbiota of the MG group was changed in terms of the relative abundances of bacterial taxa, with sharply reduced microbial richness, particularly in the genus *Clostridium*. The fecal SCFA content was significantly lower in the MG group. Furthermore, microbial dysbiosis was closely related to the levels of inflammatory biomarkers in the sera of MG patients.

## Introduction

Myasthenia gravis (MG) is an antibody-mediated, T cell-dependent autoimmune disease. It is characterized by fluctuating weakness of skeletal muscles, mainly caused by autoantibodies directed against the acetylcholine receptor (AChR) located in the postsynaptic membrane at the neuromuscular junction. The pathogenesis of MG is closely correlated with high levels of circulating AChR antibodies (Pestronk et al., 1985). Several studies have revealed that the production of AChR antibodies is associated with the disequilibrium of Th1, B cells, and Foxp3+ T regulatory (Treg) cells (Mu et al., 2009; Aricha et al., 2011). Among them, Foxp3+ CD4+ Treg cells play a critical role in maintaining self-tolerance and immune homeostasis in the prevention of the development of MG. Foxp3+ CD4+ Treg cells regulate the production of AChR antibodies by influencing the amount of autoreactive T cells and suppressing the activity of autoreactive B cells, leading to the reduction of disease severity and progression(Shin et al., 2014). In patients with MG, the frequency of Foxp3+ CD4+ Treg cells is significantly deficient and has become the major focus of many studies on the pathogenesis of MG (Fattorossi et al., 2005; Li et al., 2008; Masuda et al., 2010). Therefore, novel approaches that can restore the defects in Foxp3+ CD4+ Treg cell numbers will be valuable for the treatment of MG disease.

Foxp3+ CD4+ Treg cells are the most prominent regulatory cells in the body. The frequency of Foxp3+ CD4+ Treg cells is notably higher in colonic lamina propria than in any other organ (Atarashi et al., 2011). Furthermore, the Foxp3+ CD4+Treg cells in intestinal lamina propria have unique characteristics, which are markedly affected by the composition of gut microbiota (Nagano et al., 2012). For instance, the colonization of germ-free mice with a mix of *Clostridium* strains increases the frequency of Foxp3+ CD4+Treg cells in colonic lamina propria. In addition, a mixture of 17 strains of *Clostridium* isolated from a healthy person strongly induces Foxp3+ CD4+Treg cells in the human colon. These data provide new insights indicating that Foxp3+ CD4+Treg cells in the intestine maintain homeostasis of the gut microbiota (Nagano et al., 2012; Atarashi et al., 2013). Indeed, the gut microbiota could affect the number and T-cell receptor (TCR) repertoire of Foxp3+ CD4+ Treg cells. The TCRs on Foxp3+ CD4+ Treg cells can recognize subsets of commensal bacteria, inducing naive CD4+ T cells to differentiate into antigen-specific Foxp3+ CD4+Treg cells, leading to an increased amount of Foxp3+ CD4+ Treg cells (Cong et al., 2009; Lathrop et al., 2011). Remarkably, the frequency of Foxp3+ CD4+ Treg cells is significantly deficient in MG patients and has become the major focus of interpreting the pathogenesis of MG. Therefore, we hypothesized that perturbations in the composition of gut microbial communities may be associated with intestinal bacteria-induced Foxp3+ CD4+Treg cell deficiency. Modifying the intestinal microbiome could be crucial for the design of therapeutic interventions toward MG. However, to date, no studies have evaluated the microbiota profiles of MG patients. To address this issue, we report a case-control study and analyses of the gut microbial communities in MG patients. We also measured fecal short-chain fatty acids (SCFAs) to investigate whether specific changes in microbial composition may affect the production of microbial metabolites, which may play critical roles in regulating Treg differentiation through epigenetic modifications. According to our data, changes in microbial composition, diversity, and metabolites seem to have far-reaching effects on MG disease.

## Materials and Methods

### Human Subjects and Sample/Data Collection

Patients with MG disease (average age 43.6 ± 15.1; sex, male:female, 31:22, BMI: 22.9 ± 3.6), hospitalized at Xiangya Hospital, were recruited during a period of symptom stability (no changes to their muscle weakness and abnormal fatigability as determined by the Quantitative Myasthenia Gravis [QMG] Score). MG disease was diagnosed based on the patient’s medical history and clinical manifestations including fluctuating muscle weakness with fatigability, a positive response to cholinesterase inhibitors, and/or a decreased response to repetitive motor nerve stimulation and/or positive single fiber electromyography (Jaretzki et al., 2000). A group of age and sex-matched healthy individuals (average age 46.2 ± 12.3; sex, male:female, 31:19, BMI: 23.1 ± 3.4) recruited from the general population were included as healthy controls (Table 1). Participants with any of the following were excluded: hepatic and/or renal diseases, psychiatric disorders, neoplasia, gastrointestinal tract disorders, metabolic diseases or any other disease that could affect the results of the study. None of the individuals received a pharmacological dose of antibiotics, glucocorticoids, anti-obesity agents, monoclonal antibodies, hypoglycaemic agents, or probiotics in the 3 months prior to the study. Participants were asked to collect their first morning feces as a baseline and were required to defecate directly into the tube or pass feces into a large clean container and then immediately transfer the fecal sample into a collection tube using the scoop attached to the screw-capped container. All collected samples were preserved at 4°C during transportation (Chong et al., 2015). The fecal specimens were stored at -80°C until use. Each participant had been informed and had signed the approved consent form prior to their participation in the study. The consent form is held by the authors’ institution and is available for review. The study protocol was approved by the Human Ethics Committee of Central South University and performed according to the declaration of Helsinki.

**Table 1:** Characteristics of MG patients and healthy control.

Characteristic	MG group	Healthy control
Age (y)	43.6 ± 15.1	46.2 ± 12.3
Sex (M/F)	31/22	31/19
Height (cm)	154.1 ± 3.4	152.8 ± 4.5
Weight (kg)	54.3 ± 7.5	53.2 ± 8.2
BMI (kg/m^2^)	22.9 ± 3.6	23.1 ± 3.4
**Ethnicity**		
Han	50 (94%)	48 (96%)
Non-Han	3 (6%)	2 (4%)
Disease course (month)	0.6 ± 0.2	—
Alcohol consumption	7 (7.5%)	5 (10.0%)
Current smoker	6 (11.3%)	4 (8.0%)
**Fatfiber diet groups**		
High fat and low fiber – yes	18 (33.9%)	19 (38.0%)
High fat and low fiber – No	30 (55.6%)	23 (46.0%)
Unknown	5 (9.4%)	8 (16%)
**Clinical subtype**		
Ocular form	35 (66.1%)	—
Generalized form	18 (33.9%)	—
**QMG scores ^∗^**		
Ocular form	Ranged from 5 to 13	—
Generalized form	Ranged from 8 to 16	—
**Complicated with thymoma**		
Ocular form	7 (20.0%)	—
Generalized form	4 (22.2%)	—
**Neostigmine intake (mg/d)**		
120 mg/d	3/15 (20.0%)	—
180 mg/d	8/15 (53.3%)	—
240 mg/d	4/15 (26.6%)	—

*^∗^The QMG score consists of 13 items with a global score ranging from 0 to 39; which is obtained from ocular, bulbar, respiratory, and limb muscle function. Higher score on QMG expresses more severe myasthenic deficit.*

### DNA Extraction, Sequencing and Statistical Analyses

Total bacterial DNA was extracted from the fecal samples using the DNA Isolation Kit (MoBio, Carlsbad, CA, United States) according to the manufacturer’s protocol (Nielsen et al., 2003). Briefly, the fecal samples were diluted in phosphate-buffered saline and homogenized with an easyMIX Lab Blender. Next, all cells were treated with lysozyme for 15 min. The lysates were mixed with binding buffer and genomic DNA (gDNA) was purified using resin columns following manufacturer’s instructions (Zhang et al., 2017). For each DNA sample, the universal bacterial forward primer 334F (5′-GTGCCAGCMGCCGCGGTAA-3′) and reverse primer 912R (5′-GGACTACHVGGGTWTCTAAT-3′) were used to amplify the V4 hypervariable region of the 16S rRNA gene using the following parameters: initial denaturation at 94°C for 3 min followed by 35 cycles of 94°C for 45 s, 50°C for 60 s, and 72°C for 90 s with a final elongation for 10 min at 72°C to ensure full amplification. PCR products were selected by excising the DNA band of the correct size and purifying it using the Wizard SV Gel and PCR Clean-Up System (Promega, St. Louis, MO, United States). Sequencing was performed on a 454 GS FLX Titanium pyrosequencer (454 Life Sciences, Branford, CT, United States) at BGI-Shanghai. Raw DNA sequence data were deposited in the National Center for NCBI Sequence Read Archive^1^. After sequencing, the reads were de-multiplexed into samples according to the barcodes using the QIIME software (Denver, CO, United States) pipeline with the default parameters. Primer and barcode sequences were removed. Operational taxonomic unit (OTU) clustering was performed at a 97% identity threshold using the QIIME pipeline (Caporaso et al., 2010). The relative abundances of taxa at the phylum and genus levels were calculated. Diversity was estimated based on the Chao1 value and observed species indices calculations (alpha or within-sample diversity). Principal component analyses used the phylogeny-based unweighted Unifrac distance matrices. The differences between specific taxa were analysed using analysis of variance (ANOVA) followed by *post hoc* tests, with Bonferroni and Benjamini–Hochberg false discovery rate (FDR) corrections for multiple testing. *P*-values < 0.05 were considered significant. Linear discriminant analysis effect size (LDS) was used to identify taxa that differed between cases and controls (Segata et al., 2011). LDS combines the standard tests for statistical significance (Kruskal–Wallis test and pairwise Wilcoxon test) with linear discriminant analyses (LDAs). LDS was performed under the following conditions: the α value for the factorial Kruskal–Wallis test among classes was 0.05 and the threshold on the logarithmic LDA score for discriminative features was 2.0 (Giloteaux et al., 2016). Phylogenetic Investigation of Communities by Reconstruction of Unobserved States (PICRUSt) was applied to predict metagenome function from the 16S rRNA gene data. We associated OTUs with known bacterial genomes precalculated in PICRUSt by picking closed OTUs against the Greengenes 16S rRNA gene database (13.5). Then the resulting OTU table was normalized and used for metagenome inference of orthologs in the Kyoto Encyclopedia of Genes and Genomes (KEGG) database using PICRUSt.

### Real-Time Quantitative PCR (qPCR)

Amplifications of Clostridia 16S rRNA genes were performed in triplicate for each sample using the KAPA SYBR^®^ FAST qPCR Kit optimized for LightCycler^®^ 480 and the LightCycler^®^ 480 II instrument with primers and protocols described previously (Matsuki et al., 2002; Rinttila et al., 2004). The PCR reaction mix contained 1× KAPA SYBR FAST qPCR Master Mix, 0.2 μM each Clostridia-specific primer, and 10 ng gDNA as template. SYBR^®^ qPCR was used to quantify the species *Faecalibacterium prausnitzii* (Fprau 07 and Fprau 02) belonging to Clostridia. We designed the primers using Primer-Express v. 2.0. The selected primer target sites (Fprau 07: 5′-CCATGAATTGCCTTCAAAACTGTT-3′, Fprau 02: 5′-GAGCCTCAGCGTCAGTTGGT-3′) were tested for specificity by submitting the sequences to the Probe Match program (Sokol et al., 2009). Amplification specificity of the target gene was checked by melting curve analyses. The efficiency and reliability of PCR amplifications were calculated.

### Analyses of SCFAs in Fecal Samples

Fecal samples were collected at the baseline, transported in a cooler with ice packs and then stored at -80°C prior to being aliquoted and extracted. SCFAs in fecal samples including acetate, propionate, butyrate and valerate were detected by gas chromatography. Briefly, 1000 mg fecal sample was filtered, and supernatants were obtained by centrifugation (10,000 ×*g*, 30 min, 4°C) and filtered through 0.2 μm filters. Filtrates were mixed with ethyl butyric acid (2 mg/mL) as an internal standard and stored at -80°C until analysis. Gas chromatography was performed and the SCFA concentration was determined as described previously (James and Martin, 1952; Tangerman and Nagengast, 1996; Zhao et al., 2006).

### Markers of Microbial Translocation and Systemic Inflammation

Serum samples were collected, processed and stored as described by Volpe et al. (2014). Serum levels of endotoxin core antibody immunoglobulin M (EndoCAb-IgM) and lipopolysaccharide secretion soluble CD14 (LPS-sCD14) were measured using enzyme-linked immunosorbent assay kits from Hycult (Uden, Netherlands) and R&D (Minneapolis, MN). Interferon-γ (IFN-γ), interleukin-6 (IL-6), tumor necrosis factor-α (TNF-α), TGF-β1, and SIgA were measured using the Multi-Array system (Meso Scale Discovery, Rockville, MD, United States) (Dinh et al., 2015). Data were analyzed using SPSS version 15.0. Non-parametric (Spearman) analyses were performed to evaluate the association between inflammatory biomarker parameters and altered taxa. *P*-values < 0.05 were considered statistically significant. Continuous variables are expressed as the median (interquartile range) or mean ± SD.

## Results

Patients who fulfilled the diagnosis criteria for MG (Jaretzki et al., 2000) were enrolled in the study. The mean disease duration (from MG symptom onset) was 0.6 years. The clinical subtype of MG disease is classified based on the location of the affected muscles (ocular versus generalized) (Berrih-Aknin and Le Panse, 2014). The severity of MG disease was evaluated by the QMG Score, in which higher scores represent more severe symptoms of muscle weakness. The QMG Scores in patients with ocular MG ranged from 5 to 13 (median value: 8.7), while those in the generalized MG patients ranged from 8 to 16 (median value: 13.1). Clinical features of the disease course and treatment were collected from patients’ clinical records (Table 1).

### The Gut Microbiota Differ Between MG Patients and Healthy Controls

**FIGURE 1:**
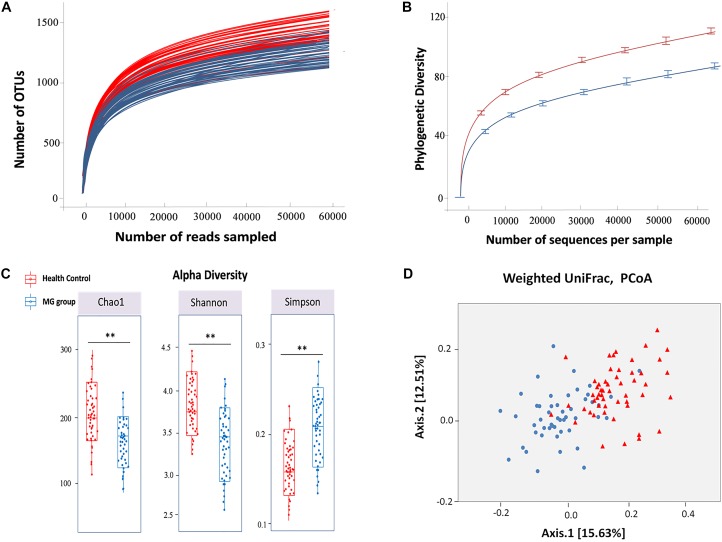
Alpha and beta diversity analysis between MG group and healthy control. **(A)** Rarefaction analysis of V4 MiSeq sequencing reads of the 16S rRNA gene in MG group and healthy controls. **(B)** The sequence-based rarefaction curves based on the Phylogenetic Diversity metric demonstrated a significant difference in the diversity of the MG group and healthy controls (*P* = 0.003, Wilcoxon rank-sum test). **(C)** Alpha diversity analysis revealed that the MG group tended to have lower bacterial evenness and richness than the healthy controls with the estimators of Chao1, Shannon and Simpson index, and Wilcoxon rank-sum test with a significance level of 0.05. **(D)** Beta diversity analysis revealed that the MG group and healthy controls were separated into 2 clusters (Weighted UniFrac distance), each triangle represents one healthy control, each dot represents one MG patient (PERMANOVA, *p* < 0.005).

The hypervariable V4 region of the 16S rRNA gene was pyrosequenced and yielded 9,672,322 high-quality sequences from all samples. After quality filtering, the mean length of the remaining sequences was 321 ± 4.1 bp, and the average number of reads/sample was 93,906 ± 20,931. In all, 97% of the sequences were assigned to a taxonomic group while 3% of the reads were unclassified. A total of 1,443 ± 507 and 1,232 ± 478 OTUs were identified in healthy control and MG cohorts, respectively, after clustering sequences at a 97% similarity threshold. Figure 1A shows the rarefaction curve at an OTU definition of 97% identity. The sequence-based rarefaction curves based on the Phylogenetic Diversity metric and the Wilcoxon rank-sum test demonstrated a significant difference in the diversity between the two groups (*P* = 0.003) (Figure 1B). Although the refraction curve increased with increases in sequencing depth, the Shannon Index had already reached a plateau and was stable for all samples (Supplementary Figure S3). These results suggest that most species were captured, although new OTUs would be expected if additional sequencing was performed. In addition, alpha diversity indices demonstrated that the MG patient group had significantly reduced microbial diversity (Figure 1C). To further evaluate the overall differences in gut microbial community structure in the MG group, we detected the beta diversity using the Bray-Curtis dissimilarity and Weighted UniFrac distances. Principal coordinates analyses (PCoA) based on weighted UniFrac measures indicated that the microbiome of the MG group was distinct from that of the healthy control (HC) group (*p* < 0.005, PERMANOVA) (Figure 1D).

**FIGURE 2:**
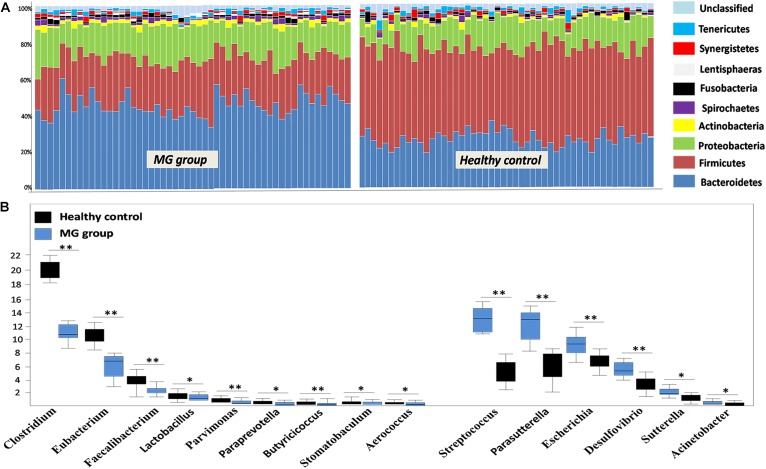
Alterations in the gut microbiome between the MG group and healthy controls at the levels of phylum and genus. **(A)** Phylum-level findings: Firmicutes was the most significantly decreased taxon in the MG group; Proteobacteria and Actinobacteria were enriched in the MG group. **(B)** Genus-level findings: the genera *Clostridium*, *Eubacterium*, and *Lactobacillus* were significantly depleted in the MG group; *Streptococcus*, *Parasutterella*, and *Escherichia* were enriched in the MG group, ^∗∗^*p* < 0.01, ^∗^*p* < 0.05.

**FIGURE 3:**
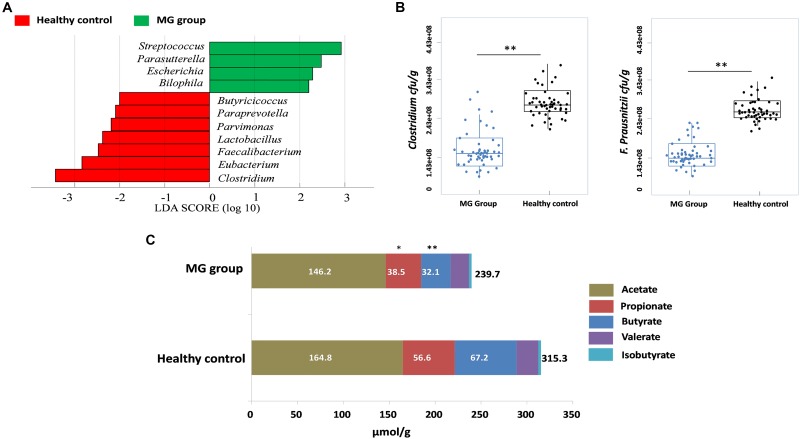
*Clostridium* was detected as the most depleted genus in the MG group. **(A)** Histogram of the LDA scores computed for genera differentially abundant between the MG group and healthy controls. Enriched genera in the MG group are indicated with a positive LDA score; enriched genera in the healthy controls have a negative score. The LDA score indicates the effect size and ranking of each differentially abundant taxon. **(B)** Absolute quantification of *Clostridium* and *Faecalibacterium prausnitzii* by means of qPCR in MG patients versus healthy controls; ^∗∗^*p* < 0.01, Wilcoxon rank-sum test. **(C)** Fecal SCFAs were lower in MG patients. The bar plot represents the median values of SCFAs in MG patients and healthy controls; ^∗^*p* < 0.05, ^∗∗^*p* < 0.01, Wilcoxon rank-sum test.

To investigate whether MG patients experienced any significant depletion of specific taxonomic groups, we applied the Wilcoxon rank-sum test to perform differential abundance analyses at the phylum levels, confining the analyses to taxa with a maximum proportion > 0.002 and prevalence > 10%. Phylum level analyses indicated that Firmicutes were significantly depleted in the MG group (*p* < 0.001), while taxa in the phyla Proteobacteria (*p* = 0.003) and Bacteroidetes (*p* = 0.002) were enriched (Figure 2A). *T*-test was applied to perform differential abundance analyses at the genus level, the relative abundances of *Clostridium* and *Eubacterium* were sharply lower in the MG group (*p* < 0.001), while the proportions of *Streptococcus* and *Parasutterella* were enriched (*p* < 0.001) (Figure 2B). LDS identified 11 discriminative features (genus level, LDA score > 2) with a significantly altered relative abundance among fecal samples taken from the two groups (Figure 3A). Because *Clostridium* was the most depleted genus, we measured the absolute amount of *Clostridium* to further validate changes in MG-related dysbiosis. qPCR analyses indicated that *Clostridium* was approximately threefold more abundant in HC than in MG (*p* = 0.002, Wilcoxon rank-sum test). At the species level, we observed that *F*. *prausnitzii* was also significantly decreased in the MG group (*p* = 0.001, Wilcoxon rank-sum test) (Figure 3B).

### SCFA Levels Are Lower in MG Patients Than in Healthy Controls

PICRUSt was used to infer the microbial metabolic pathways (Langille et al., 2013) that were enriched or depleted in the MG group. From the sequencing data, 180 KEGG pathways were identified. Among those pathways, we observed that the metabolic pathways of butanoate and propanoate were predicted to be sharply decreased in the MG group (Supplementary Figure S1). Because butyrate and propionate were the most abundant SCFAs, and these metabolites play a vital role in regulating the autoimmune system and inflammatory response (Maeda et al., 2000; Luhrs et al., 2002; Miyake et al., 2015), we calculated the fecal contents of SCFAs via gas chromatographic analyses. The overall content of SCFAs in the MG group was lower than in the HC cohort (*p* < 0.03, Wilcoxon rank-sum test), and notably, propionate and butyrate were significantly decreased (*p* < 0.05, Wilcoxon rank-sum test) (Figure 3C).

### Correlations Between Dysbiosis and Biomarkers in MG Patients

Differences in the gut microbiota composition may contribute to variability in serum biomarkers (Kim et al., 2014; Giloteaux et al., 2016). Because MG patients had distinct gut microbiota profiles, we explored how the profile changes were related to serum biomarkers. We selected the biomarkers EndoCAb-IgM and LPS-sCD14 to assess gut microbial translocation. Another five biomarkers, including IL-6 and TNF-α, were selected to assess the dysbiosis-related chronic system inflammation in MG patients. Univariate analyses revealed that the serum levels of TNF-α, IL-6 and SIgA were elevated in the MG group (*P* < 0.05), while the levels of LPS-sCD14 and EndoCAb-IgM were decreased (*P* < 0.05) (Supplementary Figure S2). Furthermore, the possible interlinks between the altered taxa and the biomarkers were explored by the Spearman method; the results are shown in Table 2.

**Table 2:** Correlation between the abundance of specific taxa and serum biomarkers.

	EndoCAb (MMU/ml)		sCD14(μg/ml)		SIgA(μg/ml)		IL-6(pg/ml)		TGF-β1(pg/ml)	
	*r*	*p*	*r*	*p*	*R*	*p*	*r*	*p*	*R*	*p*
*Eubacterium*	−0.21	0.17	0.67	<0.01	0.25	0.10	−0.19	0.15	0.13	0.16
*Lactobacillus*	0.25	0.14	−0.26	0.13	0.14	0.17	−0.21	0.06	0.12	0.17
*Clostridium*	−0.09	0.21	0.27	0.32	0.14	0.07	−0.69	0.01	0.74	0.01
*Paraprevotella*	0.17	0.34	−0.16	0.21	0.25	0.08	0.63	0.03	−0.23	0.15
*Butyricicoccus*	0.09	0.36	−0.04	0.34	0.12	0.31	−0.06	0.32	0.10	0.18
*Sutterella*	0.13	0.24	0.16	0.35	0.32	0.20	−0.16	0.19	0.41	0.06
*Desulfovibrio*	0.32	0.14	−0.27	0.25	0.19	0.24	0.36	0.03	−0.34	0.07
*Escherichia*	−0.47	0.07	0.15	0.09	0.47	0.04	−0.31	0.06	0.40	0.09
*Streptococcus*	−028.	0.19	0.68	<0.01	−0.31	0.08	−0.54	0.03	0.24	0.15

*P-values of < 0.05 are considered statistically significant.*

## Discussion

The gut microbiome may be a factor responsible for the precipitation of disease in genetically susceptible individuals (Abegunde et al., 2016; Alverdy and Luo, 2017). Our results provide the first evidence that MG disease features a dysbiotic microbiota with an overall depletion of microbial diversity, as well as an altered structure of the microbial community. Specifically, levels of commensal microbe-derived SCFAs were significantly lower in MG patients. Our results provide novel insights into MG-relevant host–microorganism interactions.

The human gut is considered a bioreactor with a high diversity of bacterial taxa, predominantly belonging to the Firmicutes and Bacteroidetes (Haro et al., 2016), that is shaped by different environmental parameters. We observed that Bacteroidetes and Firmicutes are the main bacterial phyla involved in changes in the microbiota, and the ratio of Firmicutes/Bacteroidetes (F/B ratio) in the MG patients was significantly lower than that in HC individuals. Indeed, the F/B ratio can describe a pro-inflammatory environment, the inflammatory microbiota of which might cause damage to the intestinal epithelium, subsequently triggering an immune response that contributes to the immunological imbalance characteristic of autoimmune disorders. Our results are in line with the fact that there is a shift toward a decreased F/B ratio in inflammatory bowel disease and Crohn’s disease (Andoh et al., 2011; Walker et al., 2011). Taken together, these findings lend support to the notion that changes in the F/B ratio are linked to a range of autoimmune-mediated disorders. However, its impact on the balance of anti- and pro-inflammatory forces might be more complex.

Changes in the microbial community can influence a multitude of physiological functions by regulating the host’s autoimmune system. In our study, differences between the microbiota of the HC and MG groups were identified in terms of the relative abundance of specific genera. We revealed that the richness of genera such as *Clostridium* and *Lactobacillus* were sharply decreased in the MG group. Among them, the genus *Clostridium*, a member of the Firmicutes, was the most depleted (*T*-test, *p* < 0.001). Furthermore, qPCR analyses confirmed this finding and showed that the absolute amount of *Clostridium* was approximately threefold greater in HC subjects than in MG patients. It is now well documented that specific changes in microbial composition, particularly the proportion of *Clostridium* strains, have profound effects on the number and TCR repertoire of Foxp3+ CD4+ Treg cells. Indeed, TCRs on Foxp3+ CD4+ Treg cells recognize subsets of commensal bacteria, inducing or expanding in response to bacterial stimuli. Although Clostridia do not adhere to intestinal epithelial cells (IECs), they colonize the mucus layer near the epithelium and have a powerful influence on IECs (Atarashi et al., 2011), increasing the expression of 2,3-dioxygenase and TGF-β1, which may promote the differentiation of naive CD4+ T cells into antigen-specific colonic Foxp3+ CD4+ Treg cells, consequently leading to an increased frequency of Foxp3+ CD4+ Treg cells (Cong et al., 2009; Kuhn and Stappenbeck, 2013). As an autoimmune disease, MG is characterized by the overproduction of AChR antibodies (Lindstrom et al., 1976; Engel et al., 1977); the disequilibrium of B cells and Foxp3+ CD4+ Treg cells is involved in the overproduction of AChR antibodies. Remarkably, Foxp3+ CD4+ Treg cells suppress autoreactive B cells as well as anti-AChR auto-antibody production, leading to a reduction in the severity of MG disease (Shin et al., 2014). Thus, the abundance of Foxp3+ CD4+ Treg cells is critical for the prevention of MG disease. However, the frequency of Foxp3+ CD4+ Treg cells in the peripheral blood lymphocytes is significantly deficient in MG patients. Therefore, therapeutic interventions that could restore depleted Clostridia, consequently leading to an increase in the number of Foxp3+ CD4+ Treg cells, will be novel therapeutic strategies against MG disease.

Of particular significance is the dramatically increased level of *Streptococcus* in the MG cohort. Recent studies have revealed that *Streptococcus* have a direct impact on the peroxisome proliferator-activated receptor (PPARγ) (Jones, 1988). PPARγ is involved in the regulation of immune cell proliferation and differentiation (Couvigny et al., 2015). For instance, PPARγ induces the differentiation of Foxp3+ CD4+ Treg cells, which indicates that PPARγ could lead to greater numbers of Foxp3+ CD4+ Treg cells (Nettleford and Prabhu, 2018). *Streptococcus* have been shown to regulate both PPARγ and its ligand 15d-PGJ2; the mechanism is through activation of PPARγ by inhibiting certain pathways or immune cell functions (Nettleford and Prabhu, 2018). A sharply greater abundance of *Streptococcus* has also been observed to ameliorate rheumatoid arthritis, experimental IBD, and eosinophilic airway inflammation (Kawahito et al., 2000; Hammad et al., 2004; Kim et al., 2014); our results are in line with these findings. Taken together, these findings highlight the notion that the relative abundance of *Streptococcus* can affect transcriptional regulation through factors such as PPARγ, leading to a tightly tuned balance in the immune system response (Nettleford and Prabhu, 2018).

The precise mechanism through which Clostridia regulate the differentiation and activation of immune cells remains unclear. One possible mechanism is the cooperative generation of SCFAs (Furusawa et al., 2013; Narushima et al., 2014). Microbes such as Clostridia are well-known producers of SCFAs as fermentation end-products of proteins and carbohydrates (Cummings and Macfarlane, 1991; Duncan et al., 2004). SCFAs have profound effects on T cells and directly regulate their differentiation into Foxp3+ CD4+ Treg cells (Maslowski et al., 2009; Atarashi et al., 2013). SCFAs facilitate Foxp3+ CD4+ Treg cell differentiation by at least two different mechanisms. First, exposing naive CD4+ T cells to SCFAs enhances the acetylation status of histone H3 in the promoter and CNS3 enhancer regions of the Foxp3 gene loci, leading to the differentiation of Foxp3+ CD4+ Treg cells (Furusawa et al., 2015). Second, SCFAs alter the phenotype of dendritic cells (DCs), inducing Raldh1 expression in DCs to promote the production of RA, inducing the differentiation of Foxp3+ CD4+ Treg cells (Atarashi et al., 2013). Therefore, changes in the specific microbial composition changes the production of microbial metabolites, which seem to have far-reaching effects on immunity and may be particularly relevant in the context of MG disease. Here, we observed that the levels of SCFAs were significantly lower in MG individuals. Furthermore, the abundance of Clostridia, identified as the main producers of SCFAs (Cummings and Macfarlane, 1991), was also lower in that cohort. Taken together, these results indicate that it is certainly plausible that depletion of Clostridia leads to a decrease in microbial metabolites (SCFAs), which are partly associated with deficiencies of Foxp3+ CD4+ Treg cells.

It is becoming clear that dysbiosis may drive chronic inflammation, which has the greatest influence on systemic immune responses (Forsythe et al., 2007; Karimi et al., 2009). Hence, changes in certain members of gut microbial communities are always involved in biomarkers of autoimmune inflammation. One of the mechanisms proposed to explain this pro-inflammation status is microbe translocation (MT) from an inflamed gut. To date, a widely used measure of MT is the serum concentration of lipopolysaccharide (LPS) and endotoxin core antibody immunoglobulin M (EndoCAb-IgM). Both reflect MT-associated immune activation (Monnig et al., 2016). Previous studies have reported that MT is an exclusive pathogenic characteristics in IBD/IBS (Abdul et al., 2016), graft-versus-host disease (Eriguchi et al., 2012) and HIV disease (Yim et al., 2009), which feature increased levels of LPS-sCD14 and EndoCAb-IgM. Indeed, the commensal flora operate synergistically with the intestinal barrier, and interact with the innate immune system well. Once the specific microbial composition changes, microbes as well as their metabolites are able to invade and pass through the intestinal barrier, evade immune intervention, and egress into circulation, consequently leading to immune activation and chronic system inflammation (Yim et al., 2009). In contrast to previously published data, we found that the concentrations of LPS-sCD14 and EndoCAb-IgM were significantly lower in the MG cohort, which indicates little causality between MT and the related inflammation in those patients. However, other inflammatory biomarkers, such as TNF-α and IL-6, were detected at higher levels in MG individuals. In summary, changes in the microbial communities in MG might amplify systemic inflammation by driving the expression of inflammatory mediators rather than the pathogenesis of MT.

This study had several limitations. First, the numbers of MG patients and control cases were small, limiting the ability to analyze potential confounding factors. Second, we added potassium dichromate to the collected fecal samples. Potassium dichromate is an antiseptic and effectively kills most microbial taxa. It is a strong oxidizing agent. As facultative anaerobes are more resistant to oxidation than strict anaerobes, potassium dichromate might have selective effects on the obligate anaerobes. Therefore, our results might have been affected by a relevant bias. Finally, our study, like others conducted on humans, does not answer the question of whether dysbiosis is the consequence or cause, or both, associated with MG disease. Future work to establish the causal relationship between them is needed.

## Author Contributions

JL conceived and designed the research. DQ, ZX, XJ, JD, and LZ conducted the experiments, analyzed and interpreted the data. DQ wrote the manuscript. JL supervised the study. DQ, ZX, XJ, JD, LZ, and JL revised the manuscript.

## Conflict of Interest Statement

The authors declare that the research was conducted in the absence of any commercial or financial relationships that could be construed as a potential conflict of interest.

## Footnotes

1 https://www.ncbi.nlm.nih.gov/bioproject/PRJNA445365
