# Supplementary material for: Altered Gut Microbiota in Myasthenia Gravis
Source: Front Microbiol. 2018 Nov 9;9:2627. doi: 10.3389/fmicb.2018.02627 (PMC6241162; doi:10.3389/fmicb.2018.02627)
Supplement: Supplementary file 1 [file Presentation_1.PDF]

Altered gut microbiota in Myasthenia gravis

Dongxu Qiu, Zhiwei Xia, Jun Deng, Xiao Jiao, Lei Zhang and Jing Li*

** Correspondence: Corresponding Author:Dongxu Qiu:1007351969@qq.com*

Supplementary Figures:

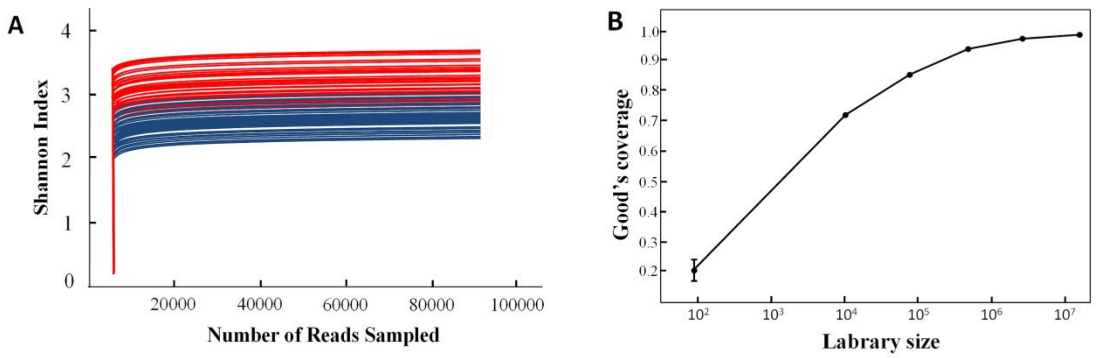

Figure S1| The Shannon index curves and the effect of library size on phylotype coverage. (A) the Shannon index reached a plateau and was stable for all the samples. (B) Randomly subsampled libraries were drawn from combined libraries and used to calculate Good's coverage estimates.

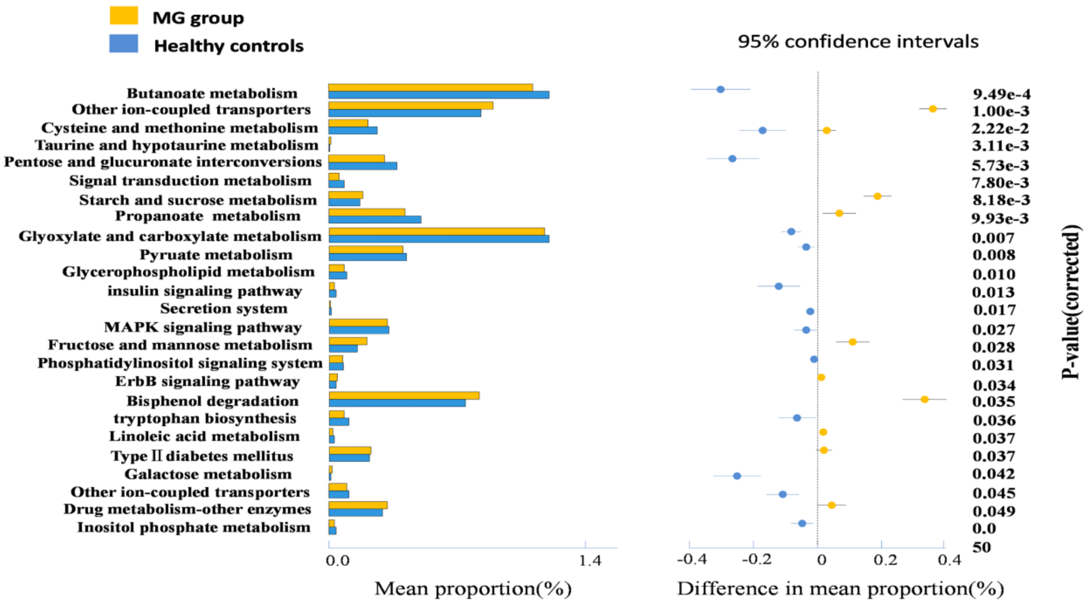

Figure S2| Predicted functional differences between MG group and healthy control. A total of 25 metabolic pathways differed significantly between cases and controls. Pathways that were more abundant in MG cases are on the positive side (orange circle with 95% confidence interval). Pathways that were more abundant in controls are on the negative side (bule circle with 95% confidence interval).

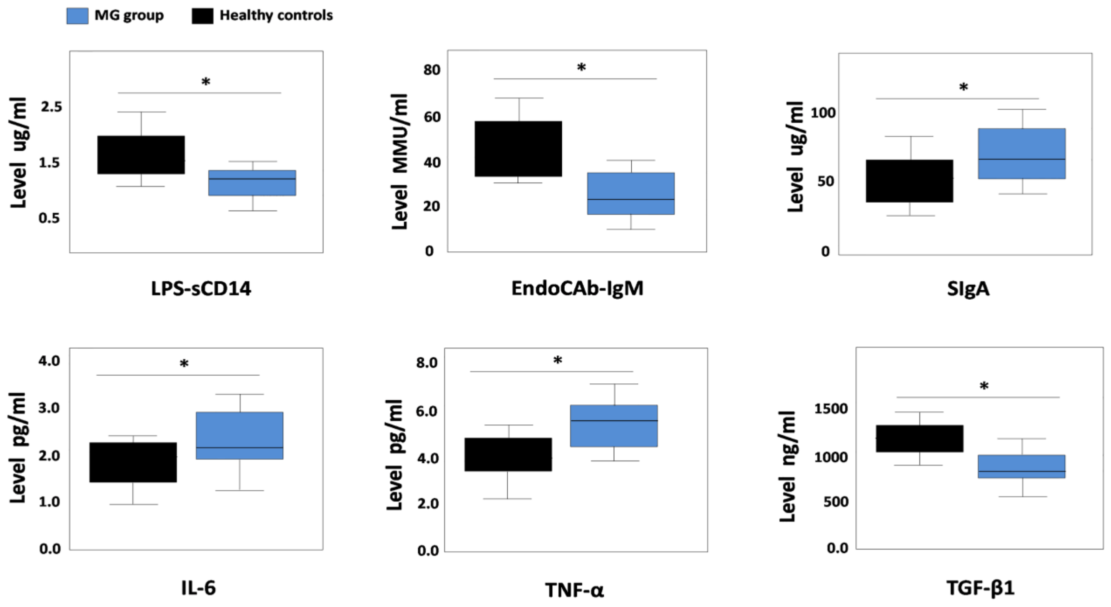

Figure S3| Markers of microbial translocation and systemic inflammation in MG group and healthy control. Levels of markers of microbial translocation (sCD14, EndoCAb, 16S rRNA gene) and systemic inflammation (IL-6, SIgA, IFN-γ, TNF-α, TGF-β1) were measured, and compared using the Mann–Whitney test, * ($p < 0.05$).
